# Paramedics' views on their seizure management learning needs: a qualitative study in England

**DOI:** 10.1136/bmjopen-2016-014024

**Published:** 2017-01-09

**Authors:** Frances C Sherratt, Darlene Snape, Steve Goodacre, Mike Jackson, Mike Pearson, Anthony G Marson, Adam J Noble

**Affiliations:** 1 Department of Psychological Sciences, University of Liverpool, Liverpool, UK; 2 School of Health and Related Research, University of Sheffield, Sheffield, UK; 3 North West Ambulance Service NHS Trust, Bolton, UK; 4 Department of Molecular and Clinical Pharmacology, University of Liverpool, Clinical Sciences Centre, Liverpool, UK; 5 Aintree Health Outcomes Partnership, University of Liverpool, Clinical Sciences Centre, Liverpool, UK

**Keywords:** ACCIDENT & EMERGENCY MEDICINE, EDUCATION & TRAINING (see Medical Education & Training), QUALITATIVE RESEARCH

## Abstract

**Introduction:**

The UK ambulance service often attends to suspected seizures. Most persons attended to will not require the facilities of a hospital emergency department (ED) and so should be managed at scene or by using alternative care pathways. Most though are transported to ED. One factor that helps explain this is paramedics can have low confidence in managing seizures.

**Objectives:**

With a view to ultimately developing additional seizure management training for practicing paramedics, we explored their learning needs, delivery preferences and potential drivers and barriers to uptake and effectiveness.

**Design and setting:**

Semistructured interviews were conducted with a purposive sample of paramedics from the English ambulance service. Interviews were transcribed and thematically analysed.

**Participants:**

A diverse sample of 19 professionals was recruited from 5 different ambulance NHS trusts and the College of Paramedics.

**Results:**

Participants said seizure management was neglected within basic and postregistration paramedic training. Most welcomed additional learning opportunities and identified gaps in knowledge. This included how to differentiate between seizure types and patients that do and do not need ED. Practical, interactive e-learning was deemed the most preferable delivery format. To allow paramedics to fully implement any increase in skill resulting from training, organisational and structural changes were said to be needed. This includes not penalising paramedics for likely spending longer on scene.

**Conclusions:**

This study provides the first evidence on the learning needs and preferences of paramedics regarding seizures. It can be used to inform the development of a bespoke training programme for paramedics. Future research should develop and then assess the benefit such training has on paramedic confidence and on the quality of care they offer to seizure patients.

**Strengths and limitations of this study**

- This is the first study to explore paramedics' views on seizure management training needs and to ascertain potential barriers and drivers to training implementation.
- The study sample includes paramedics from English NHS ambulance trusts only; however, many of the issues raised might be generalisable to other countries that have similarly organised emergency care systems—such as the USA, Australia, Canada and New Zealand.
- Our study is based on the perceptions and experiences of a self-selecting sample of participants. It also remains to be established whether addressing the training needs identified by participants would ultimately change practice.

## Introduction

Almost all patients attending UK emergency departments (EDs) for seizures have been transported there by an emergency ambulance.^1^ While once viewed as largely a transportation service, expectations of the ambulance service, like in other countries, have evolved over the last decade.^2^ Specifically, paramedics—as registered health professionals on board most ambulances—are not obliged to transport every patient to ED, but rather, should determine the most appropriate care for each patient they attend. The expectation is that the service will increasingly limit demand on EDs and associated services by using alternative, non-emergency care pathways for patients when appropriate.^3–5^

For some seizures, such as a first seizure, the paramedic would be expected to execute rapid transportation of the patient to hospital, so they can access the full facilities of an ED.^6–8^ If a prolonged or repeated seizure occurs, emergency medication may need to be administered. However, the most common seizure presentation attended to by ambulances in the UK is a person with an established epilepsy diagnosis who has experienced an uncomplicated seizure.^9^ ^10^ In contrast to many presentations paramedics encounter, such persons do not need the full facilities of an ED, but typically only reassurance and supervised rest.^6–8^

Evidence now indicates that some UK paramedics do not feel sufficiently prepared to perform the ‘see, treat and triage’ role when presented with seizures.^9^ ^11–13^ One study, for instance, examined records of one regional ambulance service's management of seizure patents.^9^ Self-termination of the suspected seizure and resumption of breathing occurred before the emergency vehicle arrived in most cases. In only 8% of cases was emergency medication needed. Despite this, ambulance clinicians still advised transport to ED in 89% of the cases attended to. This helps explain why recent UK National Audits of Seizure Management in Hospitals found most patients attending ED for seizures have experienced uncomplicated seizures and had already diagnosed epilepsy.^14^

The reasons why paramedics do not frequently use non-conveyance options has been explored by two studies.^11^ ^13^ For both, ambulance clinicians were interviewed about their experiences of managing seizures, with the results of the latest study having been presented in an article accompanying this paper.^13^ In short, interviews revealed how paramedics in England report a range of factors, beyond clinical need, that impinge on their decisions when managing seizure patients and consequently, transportation to ED remains the default option for many.

One factor of importance to decision-making is education. Specifically, participants reported little attention was given to seizure management within basic training. Dickson *et al*^9^ reviewed calls to a regional UK ambulance service. Calls relating to suspected seizures were the seventh most common, accounting for 3.3% of all calls. Paramedics, however, report only a few hours of training on seizures.^13^ As a consequence, paramedics say they often have low knowledge and lack confidence in managing seizures. It was stated that many paramedics cannot confidently differentiate between patients in terms of care needs and are often concerned about not conveying patients to ED. Telling comments from across our studies include, “I always feel safer taking them to hospital,” “I don't mind sitting there…just convincing them to go to hospital”^11^ and “there is…this sort of anxiety if they arrive on scene and the seizure has stopped. It's a big grey area…where the patient presentation is slightly beyond what you're comfortable with you take the patient to ED because it's a sort of default”.^13^

The apparent gap between the training of paramedics and the role they are now expected to perform indicates an opportunity to enhance performance and patient care by increasing the educational support available to practising paramedics through additional seizure management training. There are over 20 000 paramedics currently registered to practice in the UK.^14^

### Study objectives

More information is required regarding paramedics' seizure management learning needs, before a seizure management training programme can be developed. Given this, the current study entailed the completion of detailed qualitative interviews with paramedics to ascertain their views on paramedics' seizure management learning needs. To help understand how this training might be best delivered, we also explored participants preferences for training delivery and asked for their views on the potential barriers and drivers to the implementation and impact of any such training programme.

## Methods

### Design

Between January and March 2016, FCS (PhD), a university-based qualitative researcher with a background in health psychology but no specialist knowledge of the ambulance service, conducted individual semistructured interviews with representatives from the ambulance service. The interviews were introduced to participants as looking to explore their views regarding seizure management and what, if any, were paramedics support needs. The interviews were aided by a topic guide. Table 1 provides an overview of the relevant sections of the guide. Such a qualitative approach was considered ideal for this hitherto, largely unexplored topic. It provided a means of studying the empirical world from the perspective of the participants, allowing them to raise what they personally regarded as important aspects and concerns, rather than these being specified in advance by us, the researchers.^15^

**Table 1:** Interview topics and example questions

Interview topics	Example questions
Professional background and experience	When did you become involved in the ambulance service? What would you say are the main challenges you perceive to face in managing seizures?
Knowledge and understanding in relation to paramedic training	What are the current mechanisms for paramedic training? What are the mandatory postqualification training requirements? What training do paramedics receive on epilepsy and seizures?
Knowledge and understanding in relation to policy and practice	Do ambulance crew members within your trust have access to a seizure protocol? What kind of structure is in place around clinical decision-making?
Seizure CPD training	In your opinion, what key components should be included in a CPD training programme in this area? What would you say is the preferred format for CPD training on seizures? In your opinion, what impact do you think additional training would have on how ambulance staff manage seizures? What would be the local challenges in delivering CPD training on seizures?

CPD, continued professional development.

Full ethical approval for the research was granted by The University of Liverpool's Institute of Psychology, Health and Society Research Ethics Committee (IPHS-1516-38). Participants were informed that they could withdraw from the study at any point, that data would be anonymised and that the results might be published in a scientific journal.

### Recruitment and setting

The data reported here come from the same participants and interviews reported on in our accompanying article.^13^ Participants were paramedics recruited from the English ambulance service. Recruitment involved a study advertisement being sent to members of the ‘National Ambulances Leads’ group which has representation from the 11 regional ambulance services operating in England. Purposive sampling was employed with members being asked to circulate the advert among their respective educational, consultant and advanced paramedic teams or similar. The intention was to recruit a diverse sample of professionals, in terms of years of service and role specialism. The College of Paramedics, the professional body for the service in the UK, was also invited to help identify a representative from its educational team to be interviewed.

Participants were ultimately recruited from five different NHS ambulance services and from the College. We aimed to recruit until data saturation was achieved, which we anticipated to equate to ∼20 participants. Data saturation was ultimately achieved on recruiting 19 paramedics and therefore, recruitment ceased at this point. Most participant interviews were conducted via telephone (n=16), with the remaining being face to face.

The services recruited from included those serving mainly urban areas, those serving mainly rural areas and those serving both. In our accompanying article, we describe the characteristics of these services compared with those from which recruitment did not occur. By way of orientation, it is important to note that most (65%) practicing paramedics in England are aged between 30 and 49 years and male (62%), with the gender difference being most pronounced within managerial positions (77%).^16^ Furthermore, paramedics in England have traditionally trained through inservice training routes provided by ambulance services—the Institute of Health and Care Development paramedic programme (IHCD).^17^ A degree-level qualification has only recently become an option.^18^

### Data analysis

Interviews were audio-recorded and transcribed verbatim. QSR International's NVivo 10 (NVivo qualitative data analysis Software [program]. 10 version, 2012) was used to organise the data and to facilitate analysis. Data analysis drew on thematic analysis techniques, informed by the work of Braun and Clarke.^19^ This approach enabled the scrutiny of data across the sample and within individuals' transcripts and professional roles. The coding frame was derived deductively and inductively with identification of pre-existing themes, underpinned by previous research, as well as identification of themes emerging from the data^20^ to identify patterns and themes related to the study objectives.

FCS led the analysis process and was supported by AJN and DS. Audio recordings and line-by-line reading of the data enabled the analysts to become familiar with the data. FCS read all transcripts, AJN read the first 10 and DS read the remaining 9. Each made notes capturing significant events and themes of interest; a process similar to ‘memo-ing’ in grounded theory.^21^ Throughout analysis, emerging findings and interpretations were verified via five research team meetings. Team discussions offered fresh insight—personal, professional and methodological—and enabled FCS to reflect on potential biases and assumptions. There has been minor editing of some of the exemplar quotes to preserve anonymity and to ensure clarity of meaning.

## Results

### Participants' characteristics

Most participants were male (n=15) and had trained via the ambulance IHCD route (n=17). Purpose sampling helped generate a sample that was diverse in both years of ambulance service and role specialty (table 2). Years of service ranged from 6 to 45 (M=20, SD=9.6) and there were participants who at the time of interview held predominantly clinical (n=9), educational (n=6) and managerial (n=4) positions within the ambulance service. The average duration of interviews was 70 min (range: 47–116).

**Table 2:** Participants' characteristics

Participant	Gender	Approximate ambulance service experience (years)	Paramedic training route	Role specialism
1	Female	10	HEI	Clinical
2	Male	18	AT	Clinical
3	Male	22	AT	Clinical
4	Female	15	AT	Clinical
5	Male	25	AT	Clinical
6	Male	14	AT	Management
7	Male	6	AT	Education
8	Male	32	AT	Management
9	Male	19	AT	Clinical
10	Male	33	AT	Education
11	Female	11	HEI	Management
12	Male	21	AT	Clinical
13	Female	8	AT	Management
14	Male	21	AT	Education
15	Male	22	AT	Clinical
16	Female	24	AT	Education
17	Male	18	AT	Clinical
18	Male	45	AT	Education
19	Male	12	AT	Education

As paramedic role titles differ between regional ambulance services in England and to protect participants' anonymity, participants titles are noted according to role specialism. This was guided by the Paramedic Career Framework developed by the UK's College of Paramedics.^22^

AT, ambulance trust; HEI, higher education institute.

### Themes

Participants discussed several key aspects regarding seizure management training: (1) current learning opportunities and unmet needs; (2) required content for training; (3) preferred delivery methods and (4) barriers and drivers to implementation. Table 3 provides an overview of these themes, along with subthemes and illustrative quotes.

**Table 3:** Prominent themes, subthemes and illustrative quotes

Theme	Subtheme	Illustrative quotes
Current learning opportunities and unmet needs	Seizure management neglected	“We're really good at dealing with respiratory disorders and we're really good at dealing with heart attacks. We've had so much focus on those conditions … I just don't think that neurological disorders people feel the same level of confidence generally” (p.1) “That's the bit that frustrates me about national guidance (referring to JRCALC), it's all very much based on conveying to emergency departments because this is the problem when obviously there's a lot of other things going on after the fit that doesn't seem to be quite so focused on” (p.15) “There certainly needs to be more training on epilepsy … I think if you took most ambulance crews today and said tell me about epilepsy, tell me what's going on, tell me about serial convulsions, tell me about status epilepticus, tell me about eclampsia … I think you would start hitting boundaries, I really do” (p.8)
	Lack of training and feedback opportunities	“Well I think first, ambulance staff don't really know what community services are available for people with epilepsy, so they use A and E as a sort of gateway if you like to access and then the doctor will refer them onto wherever they need to go … so I think it would be good if the paramedics could do some training with people who are community based” (p.1) “Because staff don't get told what happened to patients it's hard for them to find out whether they're doing the right thing…epileptic patients will be an example of that. Paramedics don't know what happens to them when they get to an ED…if they knew that the last 20 they'd taken didn't have anything done to them and were discharged home then that you know that might have changed their practice” (p.3) “For every patient we took in they could say what the outcome was and they say do you know I wouldn't of took them in if I'd of known he was going to get discharged. So maybe if there was some sort of feedback saying looking you know X amount of your patients you took to hospital, 70% of the patients you took to hospital were discharged on the same day; it begs the question, why did you take them to hospital in the first place?” (p.5)
Required content for training	Managing postictal patients	“The emphasis in the initial training whether that's done at university or within the service is around status epilepticus…convulsions that need drug intervention. Any additional training should though focus on the type of patients we are typically seeing” (p.3) I think it's actually more complex than that when people go out and they're faced with somebody who's coming out of a seizure and they don't know then what to do, what they need to check, what they need to ask to at least ensure that patients safe because that wasn't really covered (p.1)
	What constitutes appropriate conveyance?	“What's normal? And what really basically what needs to go to ED? What's the red flags? I always start with does this patient have any red flags—nice and simple” (p.15) “I think it does need to be a lot more explicit that it's not going to be erm the case that we routinely transport known epileptic patients to A&E for any other reason other than you know we've got a suspicious history or we've got a change in the pattern or we've had a trauma or something unusual has happened” (p.7)
Preferred delivery methods	Interaction	“I'm certainly more visual and generally speaking as a bunch of people we are more erm hands on” (p.13) “The more interactive … then the more transferable it is in terms of erm you know the individual drawing on it when they're making the decisions in practice” (p.4)
	Pragmatic considerations	“staff have access to err when they're on when they're on station, they have access to the intranet err and they have access err both at home and on the intranet” (p.10) “It's quite hard to sometimes for them to get to work because they work rotating shifts and the shift patterns have changed now” (p.13) “while they're waiting on a standby they could tap into an iPad or their phone and look at, look at the e-learning for help, oh there's a little module on epilepsy, yes and you do it you do it online” (p.18)
Barriers and drivers to implementation	Prioritisation for seizure management training	“So if operational pressures are particularly high and staffing's particularly low … then that training might be cancelled or delayed or put off” (p.3) “I imagine there's quite a few differences (across ambulance services) … so, if we have an area that particularly we've had issues around, … then we will try and work with erm our partners on those particular issues and set up CPD events for our staff for that” (p.11) “Our mandatory training went up to a week and then it got trimmed to three days, two days and now it's trimmed to one day” (p.2)
	Continued professional development	“Ambulance crews are crying out for it (training), they really are. If you talk to staff, the one question when I … asked them what their needs are its education and CPD opportunities” (p.8) “Some sort of CPD err certificate of some sort to just identify that they've participated within that process” (p.17) “It would be nice if it was linked in, that it gave something back i.e. you know there was some CPD opportunity for them so they actually got something tangible out of it … I would like something that was maybe linked in through the College of Paramedics” (p.8)

p, participant number.

#### Theme 1: current learning opportunities and unmet needs

Most participants said seizure management was currently ‘neglected’ within basic paramedic training and postregistration training opportunities. This was said to often lead to low seizure management knowledge and confidence among paramedics. Participants described how the lack of formal training opportunities was compounded by a lack of feedback, be it formal or informal, that paramedics typically receive on how well they manage patients. This limited the opportunity for paramedics to reflect on and change practice:

One of the big problems ambulance service staff have is that we don't really get feedback so they don't know whether they've done the right thing. If they do something and they don't hear anything back from it they assume that it was ok. Because if you've done something catastrophic or you've done something wrong then you'll soon hear! (p.1)

Several participants also highlighted how this led to paramedics not being entirely clear about what care EDs actually provided to the seizure patients they conveyed.

#### Theme 2: required content for training

There was consensus across participants in terms of the required content for additional training. It was emphasised that the course should be comprehensive in its coverage of seizures, but that it needed to address the *actual* challenges paramedics commonly face. These were said to largely centre on patients who are no longer seizing, rather than emergency seizure states. Specifically, participants said that the training should help paramedics to better understand when and how to leave patients in the community:

It should say what the ‘red flags’ are? Who needs to go and what are the indications when it's safe to leave a patient at home. (p.3)

To allow paramedics to do this in practice, it was suggested that the training needed to educate paramedics about how to assess patients' histories and to help them to be able to better differentiate between different types of seizure presentations:

Show videos of different types of seizures and ask them what's happening with the patient i.e. can you differentiate perhaps the generalised tonic-clonic seizure from a psychogenic seizure and you know what sort of signs should they be looking for, what's normal… so when they see them in practice or hear about one from a bystander they better recognise them. (p.1)

Participants noted that unique challenges came from managing different types of seizure patients and in different locations (eg, rural vs urban and public vs private locations). As such, the training would need to consider this:

Looking at an elderly patient in a bed in a nursing home having a seizure in status epilepticus is entirely different to the little 4 year old child at home having another seizure. So the training would need to go into the specifics of potentially different cases. (p.18)

To facilitate non-conveyance, participants noted that the training would need to challenge inaccurate beliefs among paramedics and concerns about adverse events and litigation. To help do this, a number of participants said the training should present evidence on the actual rates of subsequent seizures and complications in patients who have not been conveyed to ED. It was also said that the training could explicitly communicate to participants what would be regarded as a negligent way of handling a case, so the boundary between negligence and a complex case could be better delineated. Participants also recommended that the disadvantages of conveying patients with established seizure disorders to hospitals needed to be highlighted:

What actually happens to these patients we take to ED? … do they just get stuck in a waiting room for 3 hours and 59 minutes or whatever… and then and the doctor says you know, ‘how are you?’… and they get sent home or does something else happen?…that would be helpful, so paramedics know kind of why we should change our practice. (p.3)

In line with this, most suggested the training should incorporate patients' perspectives on the topic. One provided an example of how such information might be presented:

Here's ‘Bert’, he's an epileptic, he has fits all the time, he does this, this and this is how he would prefer his condition to be treated. But this is what happens with the ambulance, ‘you dragged me to hospital and what happened was I missed my carer in the afternoon and my social care was messed up’…This helps underline how taking Bert to the hospital wasn't the right thing. (p.17)

To a lesser extent, training in managing minor injuries (eg, bitten tongue) was noted as also being a useful component that could contribute to reducing conveyance rates. So that paramedics knew who to inform if they left a patient in the community after a seizure, participants said a clearer understanding was also needed of who the usual care providers of seizure patients are and how regularly patients see them.

In terms of medication, it was noted that paramedics are not specifically trained in the administration of the rescue medication midazolam. This was viewed as a barrier to effective treatment. An update on epilepsy medications was also identified as important by some participants, not least because it could help paramedics interpret the normality of seizure presentations when they manage a patient who is uncommunicative, but who is carrying medication with them.

#### Theme 3: preferred delivery methods

Participants considered the advantages and disadvantages of various ways in which the additional training could be delivered. They consistently highlighted that paramedics tend to prefer training that incorporates an interactive element and which allows them to reflect on the management of challenging cases. Simultaneously, participants highlighted a number of factors that meant delivering traditional face-to-face group training to paramedics would be challenging; not least due to rotating shift patterns and the geographically dispersed nature of the workforce. Another important reason reported was that operational pressures meant services could only release their staff for very limited amounts of time to train:

The opportunities to take somebody away from the shift for half an hour to do some training doesn't exist at the minute because we're focused entirely on achieving our performance targets… if we don't achieve the response times we don't get the money.… Our mandatory (training) got trimmed to three days, two days and now it's been trimmed to one day. (p.2)

There's a national shortage of paramedics. We're running with huge staff vacancies and that means we're struggling to provide a core service and unfortunately what you find in those situations is the first thing to get dropped is training. (p.4)

Given these parameters, if designed appropriately, training delivered via an e-learning platform was seen as the most pragmatic learning method for paramedics. It was said to provide paramedics with flexibility by allowing them to access the training materials at a convenient time and for their learning to be subsequently be tested.

Participants frequently suggested that a video recording of an interactive teaching session would provide a learning opportunity that would combine their desire to reflect on challenging cases with access to training via an e-learning platform:

You could have a traditional classroom lecture and have that posted online. What we find is our paramedics are keen to get involved but are limited with their time…but some people are quite happy to sit down with YouTube on a phone for example… this is kind of me thinking how I would want to do it. (p.2)

In terms of delivery, it was noted that training should, at least in part, be facilitated by a seizure specialist, such as a neurologist. A number of advantages to this approach were highlighted. One was the credibility it would afford.

Participants also highlighted how it would be practical for any training to be developed in line with the national clinical practice guidelines available to paramedics, as such guidelines are carried by paramedics and could provide a useful aide memoire:

Probably the best you can hope for is you know an hour session… and so trying to remember 3 months what the list of things was that we had to check before we could leave them at home might be difficult. So having something written down in the National Clinical Guidelines Pocketbook would help. (p.3)

#### Theme 4: barriers and drivers to implementation

Participants were asked whether or not a seizure management training programme would be of benefit in improving paramedics' confidence in managing seizures. The majority of participants (n=17) were of the position that training would contribute towards improved confidence in managing seizures and suggested that it could be of benefit to the service. Most of these participants did caution that several potential barriers to its uptake and impact on practice existed.

One barrier was that unnecessary conveyances were not, it was argued, currently negatively impacting on the ambulance service directly, but rather the broader health service:

If somebody said, ‘Can you write down the top 10 problems the ambulance service faces?’, it would not be transporting patients who've had seizures to ED because it's not something that is massively impacting on us. It's impacting on EDs and patients.…If we end up spending longer on scene and discharge patients that will benefit EDs but it will mean we're now under more pressure because we haven't got any more cars or ambulances to go to emergencies…it's going to move the problem from ED to the ambulance service… (p.7)

Important insights into how services prioritised what training they offered their staff were also noted. It was said that training provided was largely dictated by the organisation's priorities or focused on aspects for which funding was linked. As such, participants highlighted how it might be necessary to use local funding arrangements available within the UK health service to incentivise ambulance services to allow their staff to access the training:

CQUIN…it's an acronym that stands ‘Commissioning for Quality and Innovation’. They are kind of targets that are negotiated with trusts every year and the trusts get paid for achieving them…you could lobby to develop an epilepsy CQUIN…you could include within that, the requirement to deliver X amount of training in epilepsy…that's probably the most appropriate way to influence something around this area. (p.9)

Other drivers to implementation and uptake were identified. These included a responsibility on paramedics as registered health professionals to undertake continued professional development to maintain their ability to practice and a general willingness to learn and deliver high quality care among paramedics:

Paramedics like to see things happen and make a difference… So jobs like diabetes—we give a drug, you bring them round, they stay at home, they thank you, you refer them on, it's like ‘good job’, done. If for seizure management paramedics were able to do the same thing and define the patients care you'll find that paramedics will do it more and more…it actually gives them worth as well rather than just going to somebody, taking them to hospital and that's the last we hear of it… (p.6)

Notably, a small number of participants suggested that practice change may be limited, partly because some paramedics do not welcome the new responsibility of patient discharge. Exploring this issue further highlighted how some paramedics considered the responsibility of such a task to be unusual for professionals who were paid at the grade most paramedics are:

I think there will be some staff that could undergo training and still wouldn't change their behaviour…some will feel they're not paid or appropriately banded to make those decisions…So ‘see, treat and discharge’, which is essentially what we're talking about here…There isn't really anywhere in the health service. where staff on band 4 or 5 of the NHS pay scale would make those decisions… (p.9)

## Discussion

With a view to developing a training programme for paramedics, we conducted the first study to explore paramedics' learning needs regarding seizure management. Learning needs mostly centred on postictal seizure management. Paramedics also provided important insights into their preferences for how training on this topic should be delivered, as well as potential drivers and barriers to its uptake and impact on practice. Uniquely, we explored the views of paramedics from five different ambulance services across England.

### Opportunities for improving knowledge

As well as limited coverage of seizures within basic training, paramedics reported seizures were neglected within postregistration training opportunities. Not only was there a lack of formal learning opportunities, but also informal opportunities to reflect on and learn from one's practice. This accords with previous research that has suggested limited access to feedback generally within the ambulance service can be a barrier to individual and organisational learning and improvement.^23^

The Consolidating Framework for Implementation Research^24^ offers insights into the conditions under which new interventions are and are not likely to be successfully implemented. It highlights that a ‘tension for change’ is one condition that facilitates successful uptake and implementation, that is, stakeholders need to perceive the current situation as needing to change and so be receptive to arrival of the new intervention.^24^ An older study looking at UK paramedics' willingness to undertake professional development more generally had suggested paramedics might be resistant to invest in their own development.^25^ This would indicate that there might not be sufficient ‘tension for change’ among paramedics to ensure any new training course on seizure management would be successfully implemented. Our results, however, indicate that there is enthusiasm among much of the paramedic community for the development and provision of such opportunities regarding seizures. Our contrasting finding is clearly an important one and may be due to paramedics reporting seizures to be a particularly worrying presentation to manage.^11^

Our results highlighted gaps in paramedic knowledge, which might explain why conveyance for seizures remains so high. In particular, paramedics were keen to learn more regarding differentiating seizure types and about which patients did and did not necessitate transportation to ED. These gaps in knowledge were said to occur because basic paramedic training and practice guidance documents^26^ ^27^ focus on the management of emergency states, despite these being the least frequent presentation encountered by paramedics.^9^

Paramedics' fears and concerns about leaving patients within the community would also need to be addressed by the training programme. To help achieve this, participants said information on the actual rate of complications in the population of patients left in the community could be disseminated. Some evidence is already available on this and could be incorporated into a future training programme. Mechem *et al*^28^ followed outcomes over 72 hours, for patients who had refused hospital transportation after a seizure. They found 94% had no further seizure activity and that no deaths or serious complications were observed.

### Implications for designing an interactive e-learning programme

The interviews with participants were semistructured and participant-led—participants were asked open-ended questions regarding their views on the format of a possible seizure management training programme. The results provided insights into training delivery preferences. Paramedics said interactive and practice-based training was essential. They acknowledged, however, that face-to-face training would not be feasible. The reported lack of time available, alongside the mobile and dispersed nature of the paramedic workforce, led e-learning to be identified by participants as the most pragmatic option of delivering such training.

Almost no prior evidence is available on UK paramedics' seizure learning needs; however, our findings are reflected in research conducted elsewhere, demonstrating that paramedics prefer learning methods that provide opportunities to reflect on practice and that they are less keen on non-practical or theoretical activities.^29^ ^30^ This may explain why current participants preferred an e-learning platform but also wanted a recording of an interactive session in which challenging seizure cases and scenarios were discussed by paramedics with a specialist facilitator and for learning to be subsequently tested.

While our study provides important insights, it does nevertheless represent only an initial step towards offering further seizure training to paramedics. While the specifics of the training programme remain to be determined, it will likely comprise a ‘complex intervention’ in that it will consist of several interacting components and its ‘active ingredient’ will be difficult to precisely specify.^31^ The Medical Research Council provide guidance on the development of such interventions. They contend best practice is to develop them systematically, through literature identification, modelling of processes and theory development and then to test them using a carefully phased approach, starting with a series of pilot studies targeted at each of the key uncertainties in the design, progressing to an exploratory and then a definitive evaluation. We contend that our study provides evidence that will help with the formulation of a model to understand what the knowledge limitations of paramedics are and how these may impact on the management of patients with epilepsy.

Since our study focused in depth on a relatively small number of individuals, the next step would be to examine the generalisability of our findings by conducting a large-scale national survey. This will allow examination of whether the views and preferences of the few are supported by those of the many; and to quantify the relative weightings given to these views and preferences by a larger and more inclusive group.

Having established this and formulated a viable training package, any new training package would need to be evaluated to ensure that it meets paramedics' needs and led to the desired changes in practice. Outcome measures could include paramedic confidence and skills in seizure management and patient experience. It would also be necessary to evaluate its cost. While there would be a cost to offering the training, savings could be generated by fewer patients being subsequently transported to hospital. An indication of the magnitude of the potential savings that could be realised by an intervention if effective is given by Ridsdale *et al*.^32^ They reported that in 2008/2009, there were 37 140 NHS hospital admissions for which epilepsy was the primary diagnosis. Six out of seven admissions for epilepsy are made on an emergency, rather than planned basis.^33^ The average episode cost was estimated at £1514,^34^ which indicated a total annual inpatient cost of £56.2M.

### Potential barriers to uptake and effect

Most of those interviewed expressed enthusiasm to varying degrees for the development of seizure management training for paramedics and highlighted that its uptake could be high as paramedics in England are required to undertake continuing professional development to maintain their registration (albeit if the duration and content of it is not prescribed^35^). Participants did, however, identify several barriers that might hinder the uptake and effect of any such training programme.

One was that the behaviours the training would encourage—namely, fewer conveyances to EDs and more management of patients at the scene—do not align with the way UK ambulance services' performance is currently measured. Specifically, time-based targets have dominated ambulance service funding.^36^ Non-conveyance would likely mean longer times ‘on-scene’ and therefore, slower response to subsequent calls and consequent penalisation of the service and potentially paramedics. This would understandably limit the effect any training would have. Recently announced changes to the way ambulance services in England are monitored may, however, mean this barrier will be removed. Specifically, as of 2016–2017, service will be asked to reduce conveyance rates by ∼5% each year and there will financial incentives associated with this.

A second factor was that some paramedics considered the responsibilities associated with non-conveyance as being beyond the scope of the grade at which most paramedics are currently employed—namely, ‘Agenda for Change Band 5’, which ranges from £21 909 to £28 462 per annum.^37^ The argument that paramedic grading is outdated and not reflective of the extended scope of practice paramedics are now being asked to perform is one that has been identified more broadly and discussions are underway to increase the pay of paramedics.^37^ Whether an increase occurs remains to be seen.

A final barrier that we believe needs highlighting here as it might hamper the benefit of seizure management training is paramedic access to patients' medical histories. Our participants identified the need for any new training programme to better highlight to paramedics the ‘red flags’ in seizure management that necessitate a person being transported to ED (eg, if it was the person's first seizure, or if they had experienced a seizure of an unusual duration for them). The ability of a paramedic to use this newly acquired knowledge to change their practice would though depend on the information the paramedic had about the patient they were attending to (eg, knowing whether or not they have established epilepsy and what their usual seizure presentation was). In our accompanying paper,^13^ we reported that paramedics state that they continue to have limited access to this information when ‘on scene’ and that they do not typically have time to seek it from elsewhere (eg, general practitioners). For any new training programme to have maximum benefit, the access that paramedics have to such information would need to improve; how this can be achieved remains to be determined, although better information sharing between health sectors is one possibility. Studies from the wider literature (eg, refs 38, 39) suggest that an additional avenue that could be worthy of exploration is if ‘call handlers’ within ambulance dispatch units might be able ask additional questions about the presentation and patient when receiving calls relating to suspected seizures, so that paramedics can be more informed on arrival ‘on scene’ and be able to more efficiently identify ‘true’ emergency presentations.

### Strengths and limitations

This is the first study to explore paramedics' views on seizure management training needs and to ascertain potential barriers and drivers to training implementation. Unlike previous qualitative studies exploring paramedic practice,^11^ ^40–42^ the current project recruited participants from multiple sites across England. This increases the likelihood that the issues reported are representative of practice across the country. Although the study sample includes paramedics from only English ambulance services, many of the issues raised might be generalisable to countries with similarly organised emergency care systems, such as the USA, Australia, Canada and New Zealand.

Potential limitations include that the present study is based on the perceptions and experiences of a self-selecting sample of participants. The findings may therefore differ from actual field observations. Moreover, the views of participants drawn to take part in our study may differ in important ways from those who did not. It also, of course, remains to be established whether addressing the training needs identified by our participants would lead to practice change.

## Conclusions

This study provides the first evidence on the learning needs of paramedics in relation to seizure management, as well as providing some indication of their preferences. The findings can be used to inform the development of a bespoke training programme for paramedics. Future training programmes would need to be evaluated to ascertain benefit to paramedic confidence and quality of care offered to seizure patients.
